# Indications and Adverse Events of Toradol: Based on FDA Adverse Event Reporting System (FAERS)

**DOI:** 10.1155/prm/8822463

**Published:** 2026-04-10

**Authors:** Jiaqi Guo, Luming Wei, Yan Zhang

**Affiliations:** ^1^ Department of Orthopedics, The Affiliated Hospital of Beihua University, Jilin, Jilin, China, beihua.edu.cn; ^2^ Department of Digital Oral Diagnosis and Treatment Center, The Affiliated Stomatological Hospital of Xuzhou Medical University, Xuzhou, Jiangsu, China; ^3^ Department of Anesthesiology, Shuyang Zhongxing Hospital, Suqian, Jiangsu, China

**Keywords:** adverse events, FAERS database, pharmacovigilance, Toradol

## Abstract

**Background:**

Toradol, a potent nonsteroidal anti‐inflammatory drug, is widely used for perioperative analgesia, especially in orthopedic surgeries. Postmarketing surveillance is crucial due to its extensive clinical application. This study aimed to investigate adverse events (AEs) associated with Toradol using the FDA Adverse Event Reporting System (FAERS), analyze the association strengths of key AEs, and provide clinical references.

**Method:**

FAERS data from 2004 Q1 to 2017 Q4 were analyzed, including reports where Toradol was the primary suspect drug. Disproportionality analyses were conducted using ROR, PRR, BCPNN, and EBGM algorithms to detect safety signals.

**Result:**

Among 377 eligible reports, key AEs included immune system disorders (anaphylactic reaction, ROR = 16.28), renal and urinary disorders (oliguria, ROR = 25.57), and gastrointestinal disorders (erosive duodenitis, ROR = 231.08). Notably, 29.86% of reports were from consumers, with a lower proportion from healthcare professionals. Most AEs (73.10%) occurred within 7 days of administration; rare but severe events, including deafness and cardiorespiratory arrest, were also identified.

**Conclusion:**

Our findings, derived from hypothesis‐generating signal detection analyses, provide real‐world safety data on Toradol, highlighting high‐risk AEs and reporting biases. Enhanced vigilance and monitoring, especially in high‐risk populations, are imperative. However, these signals require confirmation through prospective, controlled studies to establish causality.

## 1. Introduction

Perioperative pain is a complex pathophysiological response induced by traumatic stress and tissue damage [1]. It not only acts as a critical barrier to patients’ early postoperative mobilization and functional rehabilitation but may also trigger the transition to chronic pain through persistent pain signal transmission, significantly increasing medical burdens and prognostic risks [2]. In orthopedic surgeries with high trauma, such as joint replacement and spinal fusion, the incidence of moderate to severe acute pain ranges from 70% to 90% [3]. Its effective management has become a core component of the enhanced recovery after surgery (ERAS) concept, directly impacting patients’ hospital stay and quality of life improvement [4].

Toradol, a potent nonsteroidal anti‐inflammatory drug (NSAID), exerts analgesic effects by selectively inhibiting cyclooxygenase (COX) activity to reduce prostaglandin synthesis [5]. It demonstrates unique advantages in perioperative analgesia for orthopedic procedures: its analgesic potency is comparable to that of moderate opioids, while reducing opioid consumption, thereby lowering the risk of adverse reactions such as respiratory depression, nausea, and vomiting [6]. Additionally, it avoids the potential negative impact of opioids on bone healing, making it an important part of multimodal perioperative analgesic regimens.

Although the short‐term analgesic efficacy of Toradol has been validated in clinical studies, the particularity of the orthopedic perioperative setting—such as patients frequently having comorbidities such as osteoporosis and osteoarthritis, and often using anticoagulants or bone metabolism‐regulating drugs—poses challenges to its safety assessment [7]. Existing clinical trials are constrained by limitations such as small sample sizes, stringent inclusion criteria, and short observation durations, which hinder the comprehensive capture of adverse event (AE) signals in specific perioperative scenarios. For instance, the actual incidence of postoperative bleeding risk and renal impairment in elderly orthopedic patients remains unclear.

Real‐world data provide critical support to address this gap. The FDA Adverse Event Reporting System (FAERS), the world’s largest database for drug safety monitoring, contains a vast number of spontaneous reports on adverse reactions associated with Toradol use, enabling systematic reflection of risk characteristics in real clinical settings [8]. This study aims to analyze the pain management value and AE associations of Toradol in clinical practice based on the FAERS database, providing hypothesis‐generating evidence for optimizing perioperative analgesic strategies. It is important to emphasize that signal detection from spontaneous reporting systems is exploratory and does not establish causality; rather, it identifies potential safety issues for further investigation in controlled studies.

## 2. Methods

### 2.1. Data Sources

The US FAERS is a globally recognized public database that contains spontaneously submitted reports of adverse drug events (ADEs) from healthcare professionals, pharmaceutical companies, patients, and other relevant parties [9]. It encompasses information such as medication errors and product quality feedback, with data updated quarterly in accordance with strict standardization protocols [10]. Additionally, it includes details on demographics, medication specifics, pharmacological characteristics, descriptions of adverse reactions, patient outcomes, report sources, treatment duration, and indications, thereby enabling a comprehensive portrayal of the full spectrum of Toradol use and subsequent AEs [11].

### 2.2. AE and Drug Identification

In the process of exploring the indications and related AEs of Toradol based on the FAERS database, report files from Q1 of 2004 to Q4 of 2017 were extracted, and the data extraction and processing methods were meticulously optimized for the specific objectives of this study. The role of Toradol in AEs was strictly classified into four categories: primary suspect drug, secondary suspect drug, concomitant medication, and interacting drug. Among them, reports where Toradol was listed as the primary suspect drug were focused on inclusion, as they suggest a potential association between the drug and AEs. AEs were coded using the Preferred Terms from the Medical Dictionary for Regulatory Activities (MedDRA), which were then mapped to system organ classes (SOCs) to achieve comprehensive classification and analysis [12].

**FIGURE 1:**
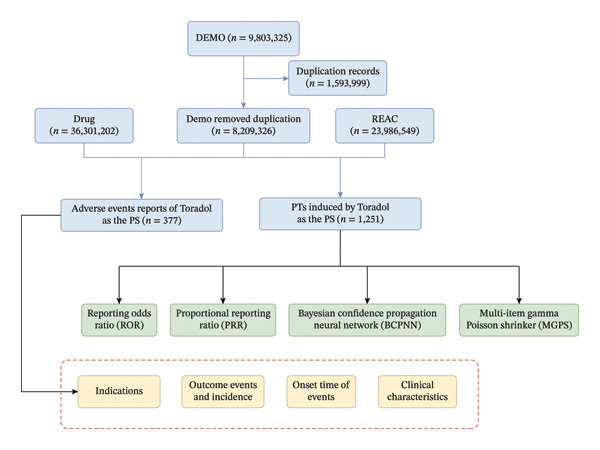
The flow diagram of selecting Toradol‐related AEs from the FAERS database.

Figure 1 outlines the key technical workflow of this study, encompassing data extraction from the FAERS database, subsequent data processing, duplicate data removal, AE classification, summarization of clinical characteristics, and disproportionality analysis between Toradol and AEs. Data processing, deduplication, and disproportionality analyses were conducted using R software (version 4.3.2) [13]. The primary analysis utilized the faers R package for structured FAERS data handling. Data visualization was created using ggplot2 R package.

### 2.3. Application of Data Analysis Techniques

To systematically identify safety signals of Toradol, this study integrated multiple disproportionality analysis methods, including reporting odds ratio (ROR), proportional reporting ratio (PRR), Bayesian confidence propagation neural network (BCPNN), and multi‐item gamma Poisson shrinker (MGPS) [14, 15]. ROR effectively corrects biases caused by small sample sizes; PRR has higher specificity than ROR; BCPNN enables integration and cross‐validation of multisource data; and MGPS demonstrates unique advantages in detecting signals of rare AEs [16]. The combined application of these complementary algorithms not only leverages the technical strengths of each method but also reduces the incidence of false‐positive results through cross‐validation and captures more potential rare adverse reactions via threshold and variance adjustments [17].

All algorithms were constructed based on 2 × 2 contingency tables to form analytical models (Table 1), with their specific calculation formulas and threshold criteria elaborated in Table 2. Higher indicator values correspond to stronger signal intensity, indicating a more robust association between the drug and AEs. In this study, systematic application of these algorithms combined with in‐depth data mining of the FAERS database enabled comprehensive and reliable identification of Toradol’s safety signals. The multialgorithm strategy not only realized cross‐validation of results but also markedly enhanced the efficiency of signal detection. It is crucial to note that the signals identified through these disproportionality analyses are statistical associations that serve to generate hypotheses; they are not substitute for causal inference derived from prospective, controlled clinical trials.

**TABLE 1:** Ratio imbalance measurement algorithm.

Item	Reports with the target AEs	All other AEs	Total
Reports with the target drug	a	b	a + b
All other drugs	b	d	c + d
Total	a + c	b + d	a + b + c + d

**TABLE 2:** Principle of disproportionality measure and standard of signal detection.

Algorithms	Calculation formula	Criteria
ROR	*R* *O* *R* = (*a*/*c*)/(*b*/*d*) = (*a* *d*)/(*b* *c*) $95\%CI=e^{\ln\left(ROR\right)\pm 1.96\sqrt{11/a+\left(1/b\right)+\left(1/c\right)+/d}}$	[1] *a* ≥ 3 [2] ROR ≥ 2 [3] 95% CI > 1

PRR	*P* *R* *R* = (*a*⁄((*a* + *b*)))/(*c*⁄((*c* + *d*))) = *a*(*c* + *d*)/*c*(*a* + *b*) *χ* ^2^ = ((|*a* *d* − *b* *c*| − *n*/2)^2^ *n*)/(*a* + *b*)(*a* + *c*)(*c* + *d*)(*b* + *d*) *n* = *a* + *b* + *c* + *d*	[1] *a* ≥ 3 [2] PRR ≥ 2 [3] *χ* ^2^ ≥ 4

BCPNN	*E*(*I* *C*) = log_2_(((*C*_*x* *y*_ + *γ*_11_)(*C* + *α*)(*C* + *β*)))/((*C* + *γ*)(*C*_*x*_ + *α*_1_)(*C*_*y*_ + *β*_1_)) *V*(*I* *C*) = 1/(*l*n2)^2^{((*C* − *C*_*x* *y*_ + *γ* − *γ*_11_)/(*C*_*x* *y*_ + *γ*_11_)(1 + *C* + *γ*)) + ((*C* − *C*_*x*_ + *α* − *α*_1_)/(*C*_*x*_ + *α*_1_)(1 + *C* + *α*)) + ((*C* − *C*_*y*_ + *β* − *β*_1_)/(*C*_*y*_ + *β*_1_)(1 + *C* + *β*))} *γ* = *γ*_11_(*C* + *α*)(*C* + *β*)/(*C*_*x*_ + *α*_1_)(*C*_*y*_ + *β*_1_) *I* *C* − 2*S* *D* = *E*(*I* *C*) − 2√((*V*(*I* *C*))) *α*_1_ = *β*_1_ = 1; *α* = *β* = 2; *γ*_11_ = 1; *C* = *a* + *b* + *c* + *d*; *C*_*x*_ = *a* + *b*; *C*_*y*_ = *a* + *c*; *C*_*x* *y*_ = *a*	[1] *a* ≥ 3 [2] IC‐2SD > 0

Abbreviations: BCPNN, Bayesian confidence propagation neural network; CI, confidence interval; IC, information component; PRR, proportional reporting ratio; ROR, reporting odds ratio.

## 3. Results

### 3.1. Fundamental Features of Toradol‐Associated ADEs

**FIGURE 2:**
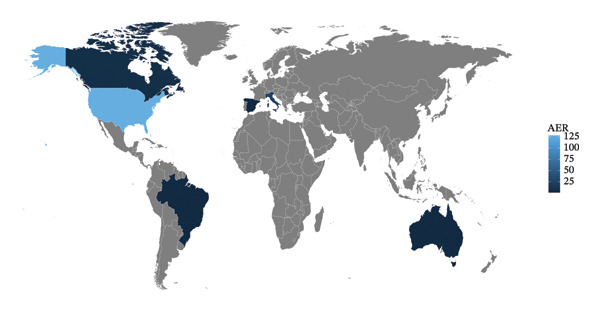
World map of AER.

This study included 9,803,325 AE reports from the FAERS database between 2004 and 2017, covering the period of Toradol’s clinical use. Among these, 377 reports identified Toradol as the primary suspect drug for ADEs. In terms of gender distribution, AE reports for Toradol showed a significant gender difference: female patients accounted for 68.17%, while male patients accounted for 31.83%, suggesting that females may be more sensitive to adverse reactions of Toradol or have a higher proportion of use in clinical practice. AE reports were more common in patients aged 18–65 years. However, approximately 26.76% of reports lacked age information, which largely limited the in‐depth and comprehensive exploration of the association between age and AEs. Analysis of report sources revealed that 29.86% of AE reports originated from consumers, reflecting active participation of consumers in monitoring drug adverse reactions. Geographically, the majority of reports (77.92%) came from the United States (Figure 2), which may be related to the scale of drug use, regulatory systems, and the maturity of adverse reaction reporting mechanisms in the country. In terms of administration routes, “other routes” accounted for the largest proportion, reaching 49.58%. For drug‐induced adverse clinical outcomes, hospitalization‐related AEs were the most prevalent, accounting for 38.24%, followed by other serious adverse reactions (28.88%), with mortality accounting for 8.82%. In terms of timing of adverse reactions, 73.10% of patients developed Toradol‐related adverse reactions within 7 days of administration, while 2.54% occurred 60 days or more after administration. The timing of adverse reactions was unknown in 15.74% of cases. Detailed information is provided in Table 3.

**TABLE 3:** Demographic characteristics of reports with Toradol from the FAERS database (2004 Q1–2017 Q4).

Variable	Total
Year	
2004	32 (9.01)
2005	28 (7.89)
2006	17 (4.79)
2007	13 (3.66)
2008	28 (7.89)
2009	23 (6.48)
2010	17 (4.79)
2011	24 (6.76)
2012	39 (10.99)
2013	35 (9.86)
2014	15 (4.23)
2015	9 (2.54)
2016	26 (7.32)
2017	49 (13.80)
Sex	
Female	242 (68.17)
Male	113 (31.83)
age_yr	51.50 (35.75,67.00)
age_yrQ	
< 18	9 (2.54)
18–45	89 (25.07)
45–65	88 (24.79)
65–75	32 (9.01)
≥ 75	42 (11.83)
Unknown	95 (26.76)
Wt	73.94 (61.29,87.09)
wtQ	
< 60	27 (7.61)
60–80	55 (15.49)
≥ 80	55 (15.49)
Unknown	218 (61.41)
Reporter	
Consumer	106 (29.86)
Physician	79 (22.25)
Other health professional	76 (21.41)
Pharmacist	45 (12.68)
Unknown	45 (12.68)
Lawyer	3 (0.85)
Registered nurse	1 (0.28)
Reported countries	
United States	120 (77.92)
Other	34 (22.08)
Route	
Other	176 (49.58)
Intramuscular	69 (19.44)
Intravenous	65 (18.31)
Oral	45 (12.68)
Outcomes	
Hospitalization	143 (38.24)
Other serious	108 (28.88)
Life threatening	46 (12.30)
Death	33 (8.82)
Disability	24 (6.42)
Required intervention to prevent permanent impairment/damage	19 (5.08)
Congenital anomaly	1 (0.27)
Tto	0.00 (0.00,3.00)
ttoQ	
< 7	144 (73.10)
7–28	12 (6.09)
28–60	5 (2.54)
≥ 60	5 (2.54)
Unknown	31 (15.74)

### 3.2. SOC

At the SOC level, AEs associated with Toradol as the primary suspect drug involved 21 organ systems. The number of AE reports and disproportionality analysis results for each system are as follows (Table 4).

**TABLE 4:** Signal intensity of ADEs of Toradol reported at the system organ classification (SOC) level in the FAERS database.

SOC	Case reports	ROR (95% CI)	PRR (95% CI)	Chisq	IC (IC025)	EBGM (EBGM05)
Immune system disorders	69	6.36 (4.99, 8.11)	6.04 (4.77, 7.64)	293	2.59 (2.25)	6.04 (4.93)
Renal and urinary disorders	49	2.6 (1.95, 3.46)	2.53 (1.92, 3.33)	46.11	1.34 (0.93)	2.53 (1.99)
Gastrointestinal disorders	196	1.91 (1.64, 2.23)	1.76 (1.53, 2.02)	70.93	0.81 (0.6)	1.76 (1.55)
Vascular disorders	41	1.38 (1.01, 1.88)	1.36 (1.01, 1.82)	4.06	0.45 (0)	1.36 (1.05)
Respiratory, thoracic, and mediastinal disorders	80	1.32 (1.05, 1.66)	1.3 (1.05, 1.61)	5.84	0.38 (0.05)	1.3 (1.07)
Ear and labyrinth disorders	7	1.28 (0.61, 2.7)	1.28 (0.61, 2.7)	0.43	0.36 (−0.65)	1.28 (0.69)
Blood and lymphatic system disorders	22	1.12 (0.73, 1.7)	1.11 (0.74, 1.68)	0.26	0.16 (−0.44)	1.11 (0.78)
Skin and subcutaneous tissue disorders	62	1.09 (0.84, 1.41)	1.08 (0.85, 1.37)	0.42	0.12 (−0.25)	1.08 (0.87)
Nervous system disorders	117	1 (0.82, 1.21)	1 (0.84, 1.19)	0	0 (−0.28)	1 (0.85)
Psychiatric disorders	70	0.93 (0.73, 1.18)	0.93 (0.74, 1.18)	0.36	−0.1 (−0.45)	0.93 (0.76)
Musculoskeletal and connective tissue disorders	62	0.9 (0.7, 1.16)	0.91 (0.72, 1.15)	0.65	−0.14 (−0.51)	0.91 (0.73)
Pregnancy, puerperium, and perinatal conditions	5	0.87 (0.36, 2.1)	0.87 (0.36, 2.1)	0.09	−0.19 (−1.35)	0.87 (0.42)
Investigations	64	0.77 (0.6, 0.99)	0.78 (0.62, 0.99)	4.22	−0.36 (−0.72)	0.78 (0.63)
Reproductive system and breast disorders	10	0.74 (0.4, 1.38)	0.74 (0.4, 1.36)	0.89	−0.43 (−1.28)	0.74 (0.44)
Cardiac disorders	29	0.7 (0.49, 1.02)	0.71 (0.5, 1.01)	3.57	−0.49 (−1.02)	0.71 (0.52)
General disorders and administration site conditions	150	0.69 (0.59, 0.82)	0.73 (0.62, 0.85)	17.49	−0.45 (−0.69)	0.73 (0.64)
Hepatobiliary disorders	8	0.67 (0.33, 1.34)	0.67 (0.34, 1.33)	1.32	−0.58 (−1.53)	0.67 (0.37)
Metabolism and nutrition disorders	19	0.66 (0.42, 1.03)	0.66 (0.42, 1.04)	3.35	−0.59 (−1.23)	0.66 (0.45)
Injury, poisoning, and procedural complications	47	0.62 (0.46, 0.83)	0.63 (0.48, 0.83)	10.72	−0.66 (−1.08)	0.63 (0.5)
Eye disorders	15	0.59 (0.35, 0.98)	0.6 (0.36, 1)	4.21	−0.75 (−1.46)	0.6 (0.39)
Infections and infestations	25	0.4 (0.27, 0.6)	0.42 (0.28, 0.62)	21.54	−1.26 (−1.82)	0.42 (0.3)

“Immune system disorders” had 69 AE reports, showing the strongest association, with a ROR of 6.36 (95% confidence interval (CI): 4.99, 8.11), PRR of 6.04 (95% CI: 4.77, 7.64), information component (IC) of 2.59 (IC025: 2.25), empirical Bayes geometric mean (EBGM) of 6.04 (EBGM05: 4.93), and chi‐square value (chisq) of 293, indicating a statistically significant association between this system and Toradol. “Renal and urinary disorders” had 49 reports, with the second strongest association: ROR = 2.60 (95% CI: 1.95, 3.46), PRR = 2.53 (95% CI: 1.92, 3.33), IC = 1.34 (IC025: 0.93), EBGM = 2.53 (EBGM05: 1.99), and chisq = 46.11, also showing a significant association signal. “Gastrointestinal disorders” had the highest number of reports (196), with ROR = 1.91 (95% CI: 1.64, 2.23), PRR = 1.76 (95% CI: 1.53, 2.02), IC = 0.81 (IC025: 0.60), EBGM = 1.76 (EBGM05: 1.55), and chisq = 70.93, indicating a certain degree of association. In addition, “vascular disorders” (41 cases) and “respiratory, thoracic, and mediastinal disorders” (80 cases) also showed certain association signals, with ROR values of 1.38 (95% CI: 1.01, 1.88) and 1.32 (95% CI: 1.05, 1.66) and PRR values of 1.36 (95% CI: 1.01, 1.82) and 1.30 (95% CI: 1.05, 1.61), respectively. IC025 was greater than 0 for both, and EBGM05 was 1.05 and 1.07, suggesting potential associations. “Ear and labyrinth disorders” (seven cases), “blood and lymphatic system disorders” (22 cases), and “skin and subcutaneous tissue disorders” (62 cases) showed weak or nonsignificant associations, with ROR and PRR values close to 1, 95% CIs including 1 for some indicators, low or negative IC025, and no significant EBGM05 signals. “Infections and infestations” had 25 reports with the weakest association: ROR = 0.40 (95% CI: 0.27, 0.60), PRR = 0.42 (95% CI: 0.28, 0.62), IC = −1.26 (IC025: −1.82), and EBGM = 0.42 (EBGM05: 0.30), indicating a weak association with Toradol. Table 4 details the number of AE reports, ROR (95% CI), PRR (95% CI), chisq, IC (IC025), and EBGM (EBGM05) for each SOC.

### 3.3. Analysis of Preferred Terms for Toradol‐Related AEs

**FIGURE 3:**
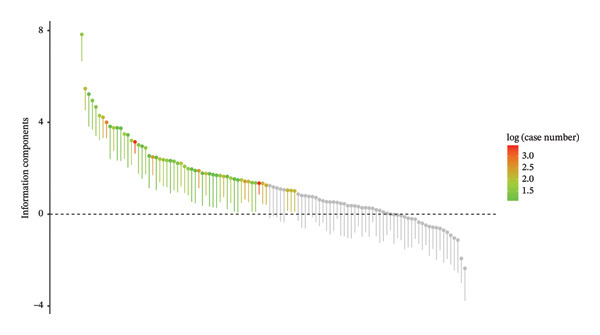
Waterfall plot of all AEs signal intensities of PT level Toradol in the FAERS database.

A series of preferred terms (PTs) was identified, with detailed information summarized in Table 5. Furthermore, a waterfall plot (Figure 3) illustrating the signal intensities of all AE signals at the PT level visually depicts the distribution of signal strengths.

**TABLE 5:** Top 30 rankings of signal intensity of Toradol adverse events by EBGM at the PT level in the FAERS database.

PT	Case reports	ROR (95% CI)	PRR (95% CI)	Chisq	IC (IC025)	EBGM (EBGM05)
Erosive duodenitis	5	231.08 (95.5, 559.11)	230.08 (95.24, 555.81)	1127.11	7.83 (6.66)	227.4 (108.57)
Duodenal ulcer	8	44.67 (22.27, 89.61)	44.36 (22.34, 88.09)	338.37	5.47 (4.52)	44.27 (24.72)
Duodenal ulcer hemorrhage	3	37.82 (12.17, 117.56)	37.72 (12.1, 117.56)	107.05	5.23 (3.82)	37.65 (14.58)
Gastritis erosive	4	31.16 (11.67, 83.23)	31.06 (11.66, 82.76)	116.18	4.95 (3.69)	31.01 (13.63)
Gastric ulcer	9	18.8 (9.76, 36.24)	18.66 (9.77, 35.63)	150.38	4.22 (3.32)	18.65 (10.77)
Melena	6	11.26 (5.05, 25.13)	11.21 (5.02, 25.04)	55.78	3.49 (2.41)	11.2 (5.72)
Esophagitis	3	10.94 (3.52, 33.97)	10.91 (3.5, 34)	27	3.45 (2.03)	10.91 (4.22)
Hematemesis	6	9.3 (4.17, 20.74)	9.25 (4.14, 20.66)	44.17	3.21 (2.14)	9.25 (4.73)
Swollen tongue	4	4.95 (1.85, 13.21)	4.94 (1.85, 13.16)	12.56	2.3 (1.04)	4.94 (2.17)
Anaphylactic reaction	16	16.28 (9.93, 26.66)	16.06 (9.84, 26.21)	226.02	4 (3.31)	16.05 (10.62)
Anaphylactic shock	7	13.67 (6.5, 28.75)	13.59 (6.45, 28.62)	81.65	3.76 (2.76)	13.59 (7.29)
Drug hypersensitivity	30	9.1 (6.33, 13.07)	8.89 (6.25, 12.65)	210.49	3.15 (2.64)	8.88 (6.56)
Hypersensitivity	13	3.73 (2.16, 6.45)	3.7 (2.14, 6.41)	25.71	1.89 (1.13)	3.7 (2.34)
Oliguria	4	25.57 (9.57, 68.29)	25.49 (9.57, 67.92)	93.99	4.67 (3.4)	25.45 (11.19)
Anuria	3	13.39 (4.31, 41.6)	13.36 (4.29, 41.64)	34.29	3.74 (2.32)	13.35 (5.17)
Hematuria	4	4.67 (1.75, 12.48)	4.66 (1.75, 12.42)	11.51	2.22 (0.95)	4.66 (2.05)
Hemiplegia	3	13.58 (4.37, 42.2)	13.55 (4.35, 42.23)	34.86	3.76 (2.34)	13.54 (5.25)
Presyncope	3	5.56 (1.79, 17.26)	5.54 (1.78, 17.27)	11.18	2.47 (1.05)	5.54 (2.15)
Dysarthria	5	5.18 (2.15, 12.47)	5.16 (2.14, 12.47)	16.79	2.37 (1.21)	5.16 (2.47)
Feeling drunk	3	14.17 (4.56, 44.01)	14.13 (4.53, 44.04)	36.59	3.82 (2.4)	14.12 (5.47)
Thirst	3	7.79 (2.51, 24.2)	7.78 (2.5, 24.25)	17.71	2.96 (1.54)	7.77 (3.01)
Swelling face	7	4.65 (2.21, 9.77)	4.62 (2.19, 9.73)	19.9	2.21 (1.21)	4.62 (2.48)
Respiratory distress	5	7.44 (3.09, 17.92)	7.41 (3.07, 17.9)	27.75	2.89 (1.73)	7.41 (3.55)
Wheezing	5	5.07 (2.11, 12.21)	5.05 (2.09, 12.2)	16.27	2.34 (1.18)	5.05 (2.42)
Postprocedural hemorrhage	6	19.72 (8.84, 44)	19.62 (8.78, 43.82)	105.94	4.29 (3.22)	19.6 (10.01)
Medication error	7	5.29 (2.52, 11.12)	5.26 (2.5, 11.08)	24.2	2.4 (1.39)	5.26 (2.83)
Hemorrhage	12	5.66 (3.2, 9.99)	5.61 (3.18, 9.9)	45.52	2.49 (1.7)	5.61 (3.48)
Fibromyalgia	4	8.16 (3.06, 21.78)	8.13 (3.05, 21.66)	25.02	3.02 (1.76)	8.13 (3.58)
Deafness	3	5.85 (1.88, 18.16)	5.83 (1.87, 18.17)	12.02	2.54 (1.13)	5.83 (2.26)
Cardiorespiratory arrest	5	4.24 (1.76, 10.21)	4.23 (1.75, 10.22)	12.32	2.08 (0.92)	4.23 (2.03)

**FIGURE 4:**
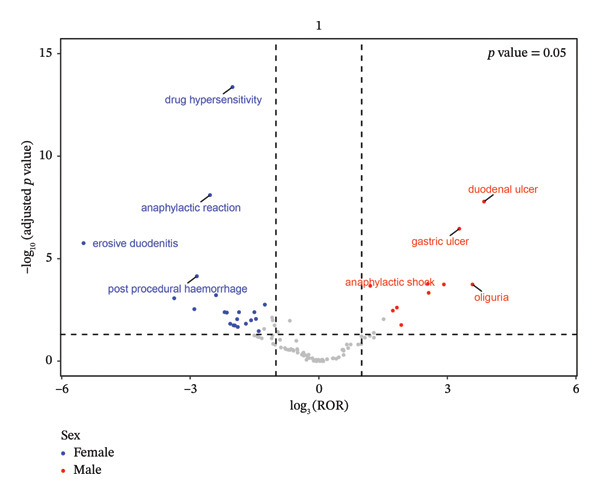
The volcano plot visualizing the magnitude of risk signals (ROR). Adverse events associated with Toradol.

After ranking by the EBGM algorithm, the top 30 PTs with the highest signal intensities were listed. Among these, AEs in multiple SOCs showed significant signal intensities. In “gastrointestinal disorders,” erosive duodenitis (five cases) exhibited prominent signal intensity: ROR = 231.08 (95% CI: 95.5–559.11), PRR = 230.08 (95% CI: 95.24–555.81), IC = 7.83 (IC025: 6.66), and EBGM = 227.4 (EBGM05: 108.57). This may be related to the pathological process by which Toradol acts on the gastrointestinal tract, disrupting the mucosal barrier and inducing local inflammatory erosion. Duodenal ulcer (eight cases) also showed strong signals: ROR = 44.67 (95% CI: 22.27–89.61), PRR = 44.36 (95% CI: 22.34–88.09), IC = 5.47 (IC025: 4.52), EBGM = 44.27 (EBGM05: 24.72), suggesting that Toradol may significantly impair the normal physiological function of the duodenal mucosa, increasing the risk of ulcers. In “immune system disorders,” anaphylactic reaction (16 cases) and anaphylactic shock (seven cases) had high signal intensities: anaphylactic reaction showed ROR = 16.28 (95% CI: 9.93–26.66), PRR = 16.06 (95% CI: 9.84–26.21), IC = 4 (IC025: 3.31), and EBGM = 16.05 (EBGM05: 10.62); anaphylactic shock showed ROR = 13.67 (95% CI: 6.5–28.75), PRR = 13.59 (95% CI: 6.45–28.62), IC = 3.76 (IC025: 2.76), and EBGM = 13.59 (EBGM05: 7.29). This indicates that Toradol, as a foreign antigen, may trigger excessive activation of the immune system, leading to severe allergic reactions that threaten patients’ lives. In “renal and urinary disorders,” oliguria (four cases) was notable: ROR = 25.57 (95% CI: 9.57–68.29), PRR = 25.49 (95% CI: 9.57–67.92), IC = 4.67 (IC025: 3.4), EBGM = 25.45 (EBGM05: 11.19), suggesting that Toradol may affect renal hemodynamics or renal tubular function, reducing urine output. Anuria (three cases), although with fewer reports, showed a certain association with Toradol use based on signal intensity: ROR = 13.39 (95% CI: 4.31–41.6), PRR = 13.36 (95% CI: 4.29–41.64), IC = 3.74 (IC025: 2.32), and EBGM = 13.35 (EBGM05: 5.17), warranting vigilance against severe renal impairment. During the study, similar to previous investigations, some AEs with low report numbers but extremely strong signal intensities were identified. For example, deafness in “ear and labyrinth disorders” (three cases): ROR = 5.85 (95% CI: 1.88–18.16), PRR = 5.83 (95% CI: 1.87–18.17), IC = 2.54 (IC025: 1.13), and EBGM = 5.83 (EBGM05: 2.26); and cardiorespiratory arrest in “cardiac disorders” (five cases): ROR = 4.24 (95% CI: 1.76–10.21), PRR = 4.23 (95% CI: 1.75–10.22), IC = 2.08 (IC025: 0.92), and EBGM = 4.23 (EBGM05: 2.03). Despite the limited number of reports on these AEs, their high signal intensities necessitate heightened vigilance regarding the potential occurrence of such rare yet severe AEs during ketorolac administration. Concurrently, Figure 4 displays a volcano plot representing all AEs associated with ketorolac, with AE signals in the upper right quadrant indicating a higher incidence during ketorolac therapy.

## 4. Discussion

As a potent NSAID, Toradol exhibits unique advantages in perioperative analgesia for orthopedic procedures [18]. Its analgesic efficacy is comparable to that of moderate opioids, while reducing opioid consumption, which largely lowers the risk of opioid‐related adverse reactions such as respiratory depression, nausea, and vomiting [19, 20]. More importantly, it avoids the potential negative impact of opioids on bone healing, making it an important component of multimodal perioperative analgesic regimens [21]. This is consistent with the short‐term analgesic efficacy validated in clinical studies and aligns with the specific requirements for analgesic drugs in the orthopedic perioperative period [21, 22]. Based on 377 relevant reports from the FAERS database (Q1 2004–Q4 2017), this study employed a hypothesis‐generating approach to analyze the application of Toradol in pain management and its associated AEs, identifying several SOCs and PTs. The safety signals discussed herein are derived from statistical associations in spontaneous reports; they are exploratory and necessitate validation through prospective, controlled studies to establish causality. From a safety perspective, orthopedic perioperative patients have unique characteristics, often with comorbidities such as osteoporosis and osteoarthritis, and frequently use combined anticoagulants and bone metabolism‐regulating drugs, making the safety assessment of Toradol more complex [23, 24].

In terms of gender distribution of AEs, female patients accounted for 68.17%, significantly higher than males (31.83%). This phenomenon may be related to gender differences in hormonal regulation of COX activity and sex‐specific expression of drug‐metabolizing enzymes (e.g., CYP450 family) [25]. In orthopedic clinical practice, female patients, especially postmenopausal women, often have comorbid osteoporosis, and their pain sensitivity and drug tolerance during the perioperative period may differ from those of males, suggesting the need for individualized analgesic regimens for female patients [26]. However, this gender‐based signal requires confirmation in studies with complete demographic and exposure data.

At the SOC level, Toradol‐related AEs involve multiple organ systems. Immune system‐related AEs showed the strongest association, with particularly high signal intensities for acute severe allergic reactions (ROR = 16.28) and anaphylactic shock (ROR = 13.67). This may be attributed to Toradol acting as a foreign substance to trigger immune responses. Additionally, immune dysfunction in orthopedic surgical patients due to traumatic stress may enhance immune responses to Toradol, while perioperative circulatory instability further amplifies the life‐threatening risks of allergic reactions [27, 28]. In clinical practice, once an allergic reaction occurs, it can rapidly endanger the patient’s life, especially in the weak state of postoperative patients, leading to more severe consequences [29]. Based on this strong signal, we recommend the following specific clinical steps to enhance vigilance: First, a thorough preadministration screening for a history of NSAID, aspirin, or other drug allergies is mandatory. Second, in settings where Toradol is administered, especially perioperatively, emergency protocols and equipment—including epinephrine, corticosteroids, and airway management tools—must be immediately available. Finally, patients should be closely monitored for signs of hypersensitivity, such as rash, wheezing, or hypotension, for at least 30–60 min after the first dose. These precautionary measures are warranted given the strength of the signal, though its causal relationship requires prospective confirmation.

Renal and urinary system AEs also showed a certain association, with high signal values for oliguria (ROR = 25.57) and anuria (ROR = 13.39), confirming the impact of Toradol on renal hemodynamics. As a key organ for drug metabolism, the kidneys in perioperative patients may experience reduced blood volume due to surgical trauma and blood loss. Additionally, elderly patients may have an age‐related decline in renal function. Toradol further reduces renal blood perfusion by inhibiting renal prostaglandin synthesis, making acute kidney injury more likely in elderly patients with hypertension or diabetes [30]. This is consistent with the general renal safety concerns of NSAIDs [31]. Therefore, for high‐risk patients—such as the elderly, those with hypertension, diabetes, pre‐existing CKD, or perioperative hypovolemia—we recommend assessing baseline renal function (serum creatinine, eGFR) before administration; avoiding or minimizing use in cases of hypovolemia; ensuring adequate hydration; and monitoring serum creatinine and urine output within the first 24–72 h of therapy, particularly with repeated dosing. The signal for oliguria reinforces the need for vigilance in this population, though prospective data are needed to quantify the precise risk.

The gastrointestinal system had the highest number of Toradol‐related AE reports, with erosive duodenitis (ROR = 231.08) and duodenal ulcer (ROR = 44.67) showing significantly higher signal intensities than other PTs. This is consistent with the pharmacological mechanism by which Toradol disrupts the gastrointestinal mucosal barrier: NSAIDs may impair the protective mechanism of the gastrointestinal mucosa by inhibiting prostaglandin synthesis, leading to mucosal damage, inflammation, and ulcers [32]. Orthopedic perioperative patients may have relatively weak gastrointestinal function due to postoperative bed rest and reduced activity, further increasing the risk of gastrointestinal AEs. Given the prominent gastrointestinal signals, proactive management is warranted. We suggest prescribing concurrent gastroprotective therapy (e.g., proton pump inhibitors) for high‐risk patients, such as those with a history of peptic ulcer, the elderly, or concurrent users of corticosteroids or anticoagulants. Patients should be educated to report symptoms such as melena, hematemesis, or severe abdominal pain promptly. For patients with significant GI risk factors, the use of alternative analgesics or formulations (e.g., topical, if appropriate) should be considered. The extreme ROR value for erosive duodenitis, while based on few cases, highlights a potential area of severe toxicity that merits this cautious approach. Notably, the significant signal for postprocedural bleeding (ROR = 19.72) suggests that Toradol may exacerbate postoperative bleeding by inhibiting platelet function. In surgical contexts where bleeding carries catastrophic risk, such as intracranial, spinal, or major reconstructive procedures, the risk–benefit ratio of Toradol should be carefully re‐evaluated, and alternative multimodal analgesia should be prioritized. In all cases, strict adherence to recommended dosing limits and duration is crucial to mitigate bleeding risk. The management strategies outlined above are based on these significant statistical signals and prudent risk mitigation.

Analysis of administration routes showed that 49.58% of AEs were associated with “other routes,” which, combined with orthopedic clinical practice, may include unconventional methods such as intra‐articular injection and local infiltration. While these routes enhance local analgesic efficacy, fluctuations in drug concentration may increase the risk of tendon damage and local tissue necrosis—studies have confirmed that topical NSAID application may inhibit tendon cell proliferation and delay tissue healing in procedures such as rotator cuff repair [33, 34]. In terms of timing distribution, 73.10% of AEs occurred within 7 days of administration, which highly overlaps with the critical window for postoperative pain management, suggesting the need for enhanced monitoring during the initiation of analgesic regimens. This study also identified previously underrecognized AE signals, such as deafness (ROR = 5.85) and cardiorespiratory arrest (ROR = 4.24). Although reports are limited (≤ 5 cases), their high signal intensities indicate potential severe risks. The mechanisms may involve Toradol‐induced electrolyte disorders (e.g., hypokalemia) and laryngeal spasm due to allergic reactions [35]. Clinicians should therefore be aware of Toradol as a potential, albeit rare, contributor to acute hearing loss and cardiorespiratory events. Standard perioperative monitoring of vital signs and oxygenation should be maintained in all patients. For those with pre‐existing cardiovascular disease or electrolyte imbalances, additional monitoring (e.g., continuous ECG, electrolyte panels) should be considered if Toradol is used. Any acute onset of these symptoms should prompt immediate evaluation and consideration of Toradol discontinuation. It is paramount to reiterate that these associations for deafness and cardiorespiratory arrest remain speculative, derived from few but high‐strength signals. They represent critical hypotheses for targeted pharmacoepidemiologic and mechanistic studies, rather than established clinical facts.

Our findings substantiate key safety warnings in Toradol’s prescribing information—notably for gastrointestinal bleeding, renal impairment, and anaphylaxis—while highlighting more specific perioperative manifestations such as erosive duodenitis. These signals reinforce essential clinical precautions: strict adherence to short‐term dosing, heightened vigilance in elderly or renally impaired patients, and cautious risk–benefit reevaluation in surgeries where bleeding risk is critical. The concentration of reported AEs within the first week postadministration further underscores the need for focused monitoring during this immediate postoperative period.

Consistent with the hypothesis‐generating nature of this study, the signals identified are generally aligned with known Toradol risks, while also highlighting potential severe but rare events that warrant further investigation. The identification of these individual severe AE signals underscores the need for enhanced review and ongoing research, specifically prospective studies designed to test the causal hypotheses generated here. These findings provide key evidence for subsequent studies and regulatory actions, aiming to ensure the safe and effective use of Toradol in clinical practice.

## 5. Limitations

This study analyzed AEs of Toradol using the FAERS database, partially addressing the limitations of traditional clinical trials with small sample sizes and short observation periods, and providing references for the real‐world safety characteristics of the drug in orthopedic perioperative applications. However, the study has the following limitations: [1] As a spontaneous reporting system, FAERS is subject to data bias and underreporting risks. Orthopedic patients often have multiple comorbidities and complex combined medications, making it difficult to fully control confounding factors, which may affect the accurate judgment of hypothesized associations between AEs and Toradol, limiting more in‐depth stratified analyses (e.g., by surgical type or comorbidity subgroup). Additionally, the database does not record the total number of exposed patients or details of treatment duration, precluding epidemiological analyses or accurate calculation of AE incidence in different clinical scenarios. [2] Voluntary reports include nonmedical professionals (29.86%), whose descriptions of adverse reactions may be subjective or contain information biases (e.g., nonstandardized recording of pain intensity and symptom duration), potentially leading to misjudgment of associations between drugs and AEs. [3] Most PTs correspond to adverse reactions already listed in drug labels (e.g., gastrointestinal damage, renal dysfunction), which may dilute the signal intensity of unlabeled events (e.g., deafness, cardiorespiratory arrest), hindering the early identification of new safety signals. [4] The disproportionality analysis methods used in this study (e.g., ROR, PRR) effectively capture potential associations but may have false‐positive risks with increasing report numbers, especially for rare but severe AEs (e.g., anaphylactic shock). Consistent with the fundamental principle of pharmacovigilance signal detection, it must be unequivocally emphasized that the signals identified in this study represent statistical associations generated from passive surveillance data. They are hypothesis‐generating in nature and cannot establish causality. Their clinical significance and causal relationships require rigorous validation through prospective cohort studies or randomized controlled trials that can control for confounding and ascertain true incidence rates. [5] Finally, as with all analyses of spontaneously reported data, confounding by indication must be considered. Toradol is primarily used for perioperative analgesia, a setting where patients are at an inherent baseline risk for several adverse outcomes due to the surgical procedure itself (e.g., bleeding, pain exacerbation, or site‐specific complications). Signals such as “postprocedural hemorrhage” or reports of increased pain may therefore reflect the underlying surgical context rather than a direct drug effect. The FAERS database lacks detailed clinical information to adequately disentangle this confounding. Consequently, the statistical signals identified in this study, especially for events common in surgical populations, should be interpreted as hypotheses that a drug‐related contribution may be present, requiring validation in studies with appropriate clinical comparators (e.g., similar surgical populations receiving different analgesic regimens) to isolate the specific effect of Toradol.

## 6. Conclusion

In summary, this hypothesis‐generating analysis based on the FAERS database provides valuable real‐world signals regarding the characteristics and potential risk associations of AEs related to Toradol. Given that 29.86% of AE reports came from consumers, whose descriptions may contain subjective biases, healthcare providers must heighten vigilance, strengthen monitoring of drug reactions, and standardize AE reporting to improve the accuracy of drug safety monitoring. Key identified AE signals include strong associations with immune system disorders (e.g., allergic reactions, ROR = 16.28), renal and urinary disorders (e.g., oliguria, ROR = 25.57), and gastrointestinal disorders (e.g., erosive duodenitis, ROR = 231.08). Additionally, rare but severe events such as deafness (ROR = 5.85) and cardiorespiratory arrest (ROR = 4.24) were flagged, requiring high clinical attention. These findings suggest the need to optimize analgesic regimens for high‐risk populations and enhance monitoring during the critical window (within 7 days of administration) to reduce risks.

The signals detected in this study are largely consistent with the known safety profile of Toradol, while newly identified rare severe AE signals must be viewed as generating important hypotheses that require validation of causality and mechanisms through prospective, controlled studies. Due to the limitations of spontaneous reporting in the FAERS database, future studies should integrate drug exposure, patient baseline characteristics, and other factors to explore risk differences in various clinical scenarios. This study provides important references for optimizing orthopedic perioperative analgesic strategies and improving drug safety supervision, emphasizing the importance of continuous monitoring and in‐depth research to balance the efficacy and safety of Toradol, ultimately ensuring the quality of patients’ postoperative rehabilitation.

## Author Contributions

Yan Zhang was responsible for the conceptualization and design of the research. Jiaqi Guo and Luming Wei carried out the data analysis. The initial draft of the manuscript was prepared by Jiaqi Guo. Yan Zhang revised the original draft. All contributors engaged in the interpretation of findings and provided critical feedback on the manuscript.

## Funding

No funding was received for this research.

## Disclosure

All authors approved the final manuscript. The findings, conclusions, and views presented here are the authors’ alone and do not necessarily reflect the FDA’s views.

## Ethics Statement

The authors have nothing to report.

## Conflicts of Interest

The authors declare no conflicts of interest.

## Supporting Information

Supporting Information 1: R code used for the analysis. This file contains the R scripts used to perform all statistical analyses and generate the figures presented in the main manuscript.

## Supporting information


**Supporting Information** Additional supporting information can be found online in the Supporting Information section.

## Data Availability

The data that support the findings of this study are available from the corresponding author upon reasonable request.
